# Neonatal Meningitis-Causing *Escherichia coli* Induces Microglia Activation which Acts as a Double-Edged Sword in Bacterial Meningitis

**DOI:** 10.3390/ijms24129915

**Published:** 2023-06-08

**Authors:** Yingying Su, Guozhen Ma, Yangyang Zheng, Jingliang Qin, Xiaoya Li, Qianwen Ge, Hao Sun, Bin Liu

**Affiliations:** 1 The Key Laboratory of Molecular Microbiology and Technology, Ministry of Education, Nankai University, Tianjin 300457, China; syyban@163.com (Y.S.);; 2 Tianjin Key Laboratory of Microbial Functional Genomics, TEDA Institute of Biological Sciences and Biotechnology, Nankai University, Tianjin 300457, China

**Keywords:** neonatal meningitis-causing *Escherichia coli*, microglia, inflammatory factor, polymorphonuclear neutrophil

## Abstract

Bacterial meningitis is a devastating disease occurring worldwide, with up to half of survivors left with permanent neurological sequelae. Neonatal meningitis-causing *Escherichia coli* (NMEC) is the most common Gram-negative bacillary organism that causes meningitis, particularly during the neonatal period. Here, RNA-seq transcriptional profiles of microglia in response to NMEC infection show that microglia are activated to produce inflammatory factors. In addition, we found that the secretion of inflammatory factors is a double-edged sword that promotes polymorphonuclear neutrophil (PMN) recruitment to the brain to clear the pathogens but, at the same time, induces neuronal damage, which may be related to the neurological sequelae. New neuroprotective therapeutic strategies must be developed for the treatment of acute bacterial meningitis. We found that transforming growth factor-β (TGF-β) may be a strong candidate in the treatment of acute bacterial meningitis, as it shows a therapeutic effect on bacterial-meningitis-induced brain damage. Prevention of disease and early initiation of the appropriate treatment in patients with suspected or proven bacterial meningitis are the key factors in reducing morbidity and mortality. Novel antibiotic and adjuvant treatment strategies must be developed, and the main goal for new therapies will be dampening the inflammatory response. Based on this view, our findings may help develop novel strategies for bacterial meningitis treatment.

## 1. Introduction

Bacterial meningitis remains a notable cause of mortality and morbidity [1]. Reports of neonatal deaths due to meningitis range from 10 to 50%, while approximately 50% of survivors sustain neurological sequelae, including seizures, hearing loss, cerebral palsy and developmental delays [2]. A major contributing factor to such mortality and morbidity is the incomplete understanding of the pathogenesis of this disease.

Certain Gram-positive and Gram-negative bacteria such as *Streptococcus pneumonia*, Group B *Streptococcus*, *Neisseria meningitides*, and NMEC can cause meningitis. NMEC (approximately 80% possessing the K1 capsular polysaccharide) is the most common Gram-negative bacterium that causes meningitis in newborns worldwide. The NMEC strain RS218 (O18:K1:H7) was isolated from the cerebrospinal fluid of neonatal meningitis patients [3,4]. This strain has been extensively used in studies related to the pathogenesis of NMEC over the past decades [3,4,5]. The development of meningitis caused by NMEC is a complex and multifactorial process. The bacteria must survive and multiply in the blood, leading to a high degree of bacteremia, which is a prerequisite for the subsequent bacterial invasion of the blood-brain barrier (BBB) [6].

Once the pathogen has reached the brain, bacteria are recognized by resident immune cells, such as microglia and astrocytes, inducing innate immune responses to the pathogen. Furthermore, circulating professional immune cells, such as PMNs, are attracted and subsequently infiltrate the infected brain parenchyma as the secretion of inflammatory factors [7]. The anti-bacterial immune response induced by the pathogen might be overwhelming and imprecisely orchestrated, leading to pronounced neuronal damage and even death [8]. As a result, survivors often sustain pathogen-specific, post-infectious sequelae, such as deafness, blindness or certain kinds of physical disability. However, the mechanisms by which the immune response occurs and induces neuronal damage are not clear.

Microglia, the innate immune cells of the myeloid lineage that reside in the central nervous system (CNS), are constantly engaged in the surveillance of their surrounding neural tissue and may contribute to health and disease in the brain [9]. However, how microglia respond to bacterial invasion and whether they contribute to neuronal damage are not clear.

Here, we determined how microglia respond to NMEC infection and the function of inflammatory factors produced by microglia that promote PMN recruitment to clear pathogens, while simultaneously inducing neuronal damage. We also found that TGF-β, a potent anti-inflammatory cytokine, may serve as an adjunctive therapy to reduce NMEC-caused, inflammation-related neuronal death and brain damage in an animal model. This may help in the development of novel strategies for bacterial meningitis treatment.

## 2. Results

### 2.1. RNA-seq Analyses of Microglia upon Infection with NMEC In Vitro

**Figure 1:**
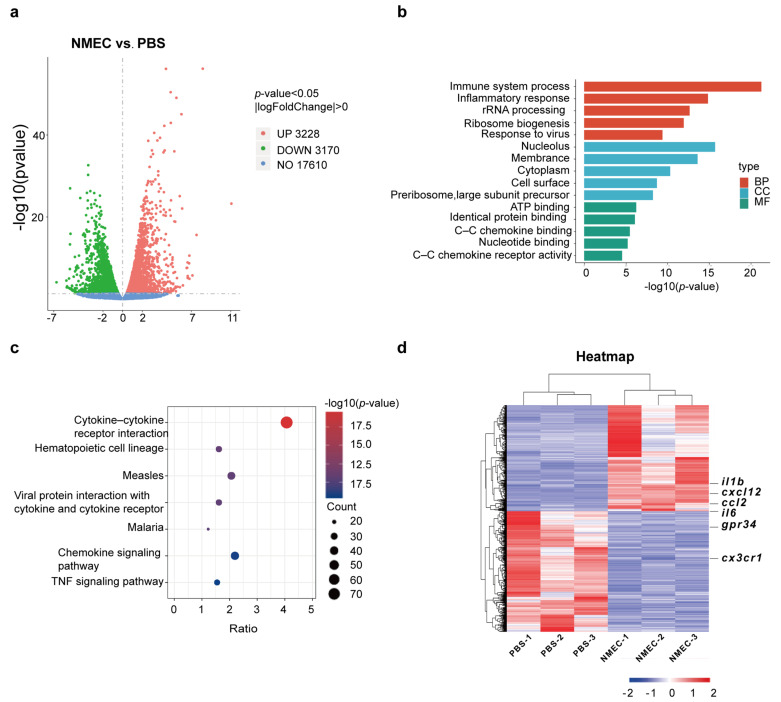
RNA-seq analysis of N9 microglial cells upon infection with NMEC in vitro. (**a**) Volcano plot showing DEGs for N9 microglial cells infected with NMEC versus PBS. Red dots represent upregulated genes; green dots represent downregulated genes. (**b**) GO enrichment analysis of DEGs includes BP, CC and MF. (**c**) KEGG terms for genes. The size of the circle represents the number of genes involved in the specific term, and the color indicates the corrected *p* values (*p* < 0.05). (**d**) Heatmap of the NMEC infection group and PBS group (n  =  3 independent experiments), with red representing higher abundance and blue representing lower abundance.

To determine the role of microglia in bacterial meningitis, we established a high-resolution transcriptome in response to NMEC infection. N9 microglial cells were infected with NMEC, and uninfected cells were used as controls. An RNA-seq transcriptional analysis was performed using three independent samples (biological replicates) of each group. DEGs between the infected and uninfected group were analyzed. A total of 6398 DEGs were identified. Compared with the uninfected group, there were 3228 upregulated genes and 3170 downregulated genes in the NMEC-infected group (Figure 1a). The top 500 genes that were significantly differentially regulated in microglia infected with NMEC are listed in Table S1 based on fold difference. Next, we performed functional classification analyses of the upregulated genes (*p* < 0.05, and |log_2_ (fold change)| > 0) using DAVID Informatics Resources through classification into the biological process (BP), cellular component (CC) and molecular function (MF) GO categories (FDR < 0.05). The DEGs were significantly enriched in the immune system process and inflammatory response in BP; nucleolus, membrane and cytoplasm in CC; and ATP binding, identical protein binding and C–C chemokine binding in MF. More detailed GO enrichment analysis results are shown in Figure 1b. KEGG pathway analysis showed that the upregulated DEGs were significantly enriched in the cytokine–cytokine receptor interaction and chemokine signaling pathway, indicating that, in response to stimuli, microglia were activated to secrete pro-inflammatory factors (Figure 1c). The heatmap shows that there was a significant difference between the infected and uninfected groups. Many pro-inflammatory genes such as *il6* and *il1b*, which are regarded as M1 phenotype gene markers, were upregulated in the infected group, which suggests the microglia may be activated towards the M1 phenotype (Figure 1d). To confirm the RNA-seq results, we performed qRT-PCR experiments and found that, compared with the uninfected group, the expressions of pro-inflammatory genes including *il6* and *il1b* were significantly higher in the NMEC-infected group, which was consistent with the results of RNA-seq analysis (Figure S1).

### 2.2. RNA-seq Analyses of Microglia Isolated from Mice That Received NMEC In Vivo via the Tail Vein

**Figure 2:**
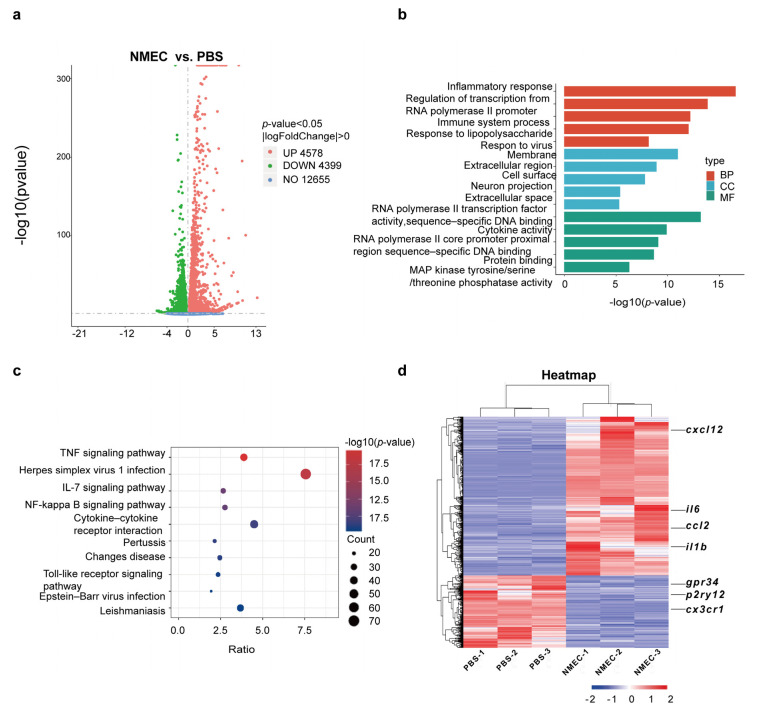
RNA-seq analyses of microglia upon infection with NMEC in vivo. (**a**) Volcano plot showing DEGs for mice that received NMEC versus PBS. Red dots represent upregulated genes; green dots represent downregulated genes. (**b**) GO enrichment analysis of DEGs included BP, CC and MF. (**c**) KEGG terms for genes. The size of the circle represents the number of genes involved in the specific term, and the color indicates the corrected *p* values (*p* < 0.05). (**d**) Heatmap of the NMEC infection group and PBS group (n  =  3 independent experiments), with red representing higher abundance and blue representing lower abundance.

To further confirm the role of microglia in penetration of the brain by NMEC, we performed RNA-seq analyses in the experimental mouse model of hematogenous meningitis. Each animal received 1 × 10^7^ CFUs of NMEC via the tail vein, and four hours later, brain specimens were collected for the isolation of microglia. DEGs between microglia isolated from NMEC-infected mice and uninfected mice were analyzed. A total of 8977 DEGs were identified. Compared with microglia from uninfected mice, there were 4578 upregulated genes and 4399 downregulated genes in microglia from NMEC-infected mice (Figure 2a). The top 500 genes that were significantly differentially regulated in microglia from NMEC-infected mice are listed in Table S2 based on fold difference. The genes were grouped into several categories based on their BP and molecular GO functions, and heatmaps were generated to aid the visualization of the gene expression pattern. We found that upregulated DEGs were significantly enriched in BP, including the inflammatory response and the immune system process (Figure 2b,c). The heatmap showed that there was a significant difference between the NMEC infection and control groups (Figure 2d). Many pro-inflammatory genes such as *il6*, *il1b*, *ccl2* and *cxcl12* were upregulated and most homeostatic genes such as *gpr34*, *p2ry12* and *cx3cr1* were downregulated in microglia from NMEC-infected mice. These results indicate that NMEC induces microglia activation towards the M1 phenotype and results in the production and secretion of pro-inflammatory cytokines, results which are consistent with the in vitro data. To verify the expression of the above genes, we performed a qRT-PCR experiment and found that compared with the uninfected group, the expression of pro-inflammatory genes (*il6*, *il1b*) was significantly higher in microglia from NMEC-infected mice (Figure S2).

### 2.3. NMEC Promotes IL-6 Secretion and PMN Chemotaxis Which May Affect NMEC Survival

**Figure 3:**
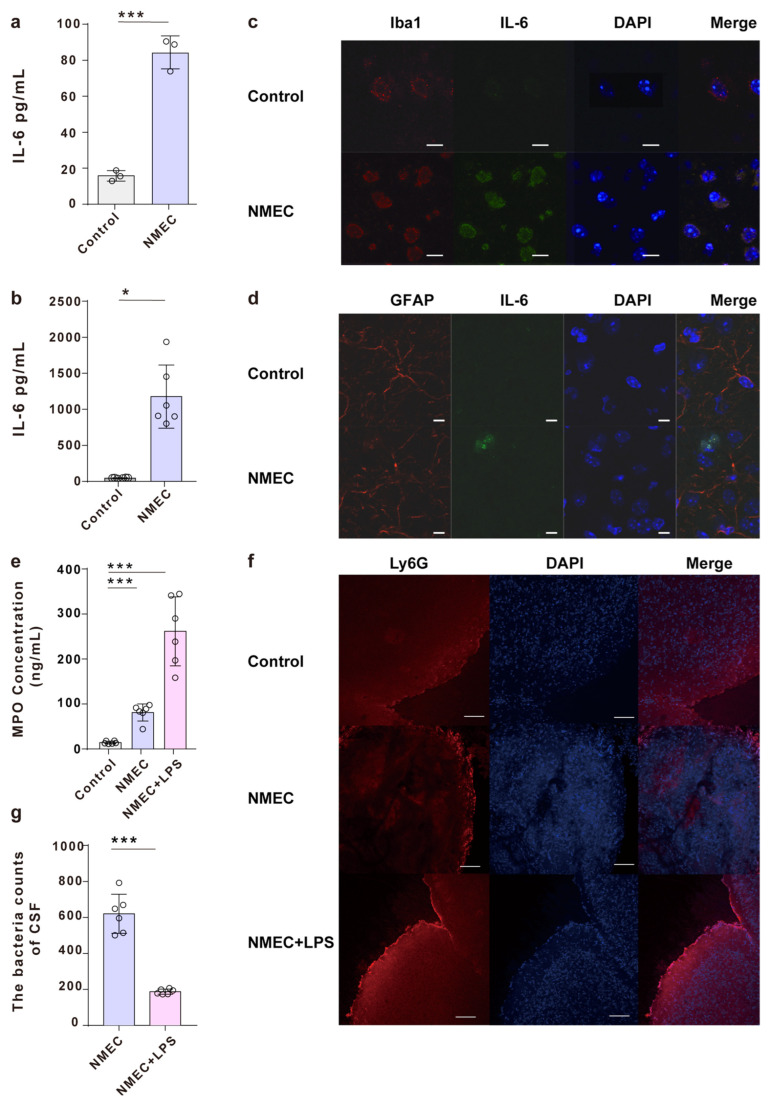
NMEC promotes IL-6 secretion and PMN chemotaxis. (**a**) ELISA quantification of IL-6 levels in microglia infected with NMEC. (**b**) ELISA quantification of IL-6 levels in CSF from mice that intracranially received NMEC or PBS. (**c**) Representative immunofluorescence images of colocalization between microglia marker Iba1 (red) and IL-6 (green) in coronal brain sections from mice that received PBS or NMEC via the tail vein. Scale bar, 10 μm. (**d**) Representative immunofluorescence images of colocalization between astrocyte marker GFAP (red) and IL-6 (green) in coronal brain sections from mice that received PBS or NMEC via the tail vein. Scale bar, 10 μm. (**e**) Quantification of MPO levels in the CSF from mice that intracranially received PBS and NMEC with or without LPS. (**f**) Representative immunofluorescence images of Ly6G in coronal brain sections from mice that received PBS and NMEC with or without LPS via the tail vein. Scale bar, 50 μm. (**g**) The number of bacteria in CSF from mice that intracranially received NMEC and NMEC with LPS. Data are presented as mean ±  SD. *n*  =  3 independent experiments a, c, d, f, *n*  =  6 b, e, g mice per group. *p* values were determined using the two-tailed unpaired Student’s *t*-test (**a**) or Mann–Whitney U test (**b**,**e**,**g**). * represents *p*  < 0.05, *** represents *p*  < 0.001.

To prove that microglia produce inflammatory factors at the protein level, the release of inflammatory factor IL-6 was evaluated via ELISA in microglia infected with NMEC in vitro (Figure 3a) and in the infected brains of mice in vivo (Figure 3b). We found that the levels of IL-6 were significantly higher in the infected group of microglia compared to the uninfected group (Figure 3a) and in the brains of NMEC-infected mice compared to the brains of mice that received PBS (Figure 3b). Since microglia and astrocytes are the two main immune cells in the brain, and they are major sources of inflammatory factors [10], we tested whether microglia are the main cells in the brain that produce IL-6 in response to NMEC infection. Immunofluorescent staining of the brain cortex slices from NMEC-infected mice revealed that microglia were colocalized with IL-6, while astrocytes were not. Therefore, the secretion of inflammatory factor IL-6 is related to the microglia (Figure 3c,d).

To identify the function of inflammatory factors during NMEC infection, we analyzed PMN recruitment to the brain. Neutrophil enzyme myeloperoxidase (MPO), which serves as an effective indicator of neutrophil infiltration [11], was significantly higher after mice received NMEC or NMEC with LPS compared with mice that received PBS (Figure 3e). In addition, the MPO levels were significantly higher after mice received NMEC with LPS, which caused higher IL-6 production than in mice that received NMEC alone (Figure S3 and Figure 3e). Similar results were observed when assessing PMN recruitment directly in the CNS. Mice that received NMEC exhibited less PMN infiltrate compared with mice that received NMEC with LPS and more PMN infiltrate compared with mice that received PBS (Figure 3f).

As recruitment of PMNs may be beneficial for clearing bacteria [12]. We administered approximately 2.5 × 10^3^ CFUs of NMEC with or without LPS via intracranial injection to identify the effect on bacteria survival in CSF, and the LPS caused more PMN infiltration (Figure 3f). After 6 h, the strain numbers were significantly lower in mice that received NMEC with LPS than those in the CSF of mice that received NMEC alone (Figure 3g), suggesting that inflammatory factors produced by microglia are associated with PMN recruitment and host defense against bacteria.

### 2.4. Inflammatory Responses Induced by NMEC Cause Neuronal Damage

**Figure 4:**
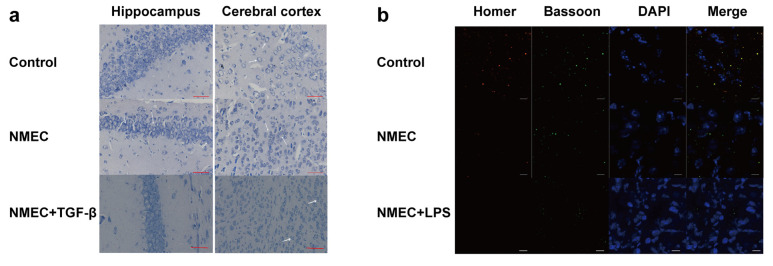
Neuronal damage caused by NMEC-induced inflammatory responses. (**a**) Nissl staining in the hippocampus and cerebral cortex of mice that received PBS and NMEC with or without TGF-β via the tail vein. Arrows show the shrunken neurons with pyknotic nuclei. Scale bar, 50 μm. (**b**) Representative immunofluorescence images of colocalization between Homer (red) and Bassoon (green) in coronal brain sections from mice that received PBS and NMEC with or without LPS via the tail vein. Scale bar, 10 μm.

In order to further identify the bacterial-meningitis-induced brain damage caused by NMEC, we performed a histological examination involving Nissl staining to assess neuronal loss [13]. More shrunken neurons with pyknotic nuclei were found in NMEC-infected mice compared with mice that received PBS (Figure 4a). The colocalization of Bassoon and Homer, which are regarded as pre- and postsynaptic proteins [14], was significantly lower in NMEC-infected mice compared with mice that received PBS (Figure 4b). These results suggest that NMEC infection triggers the host immune response to the release inflammatory factors, and then contributes to brain damage.

Previous studies have shown that TGF-β can induce microglia shift toward the M2 phenotype, which secretes anti-inflammatory cytokines and nutrient factors that promote repair and regeneration functions and restore homeostasis [15]. Recently, the therapeutic effects of TGF-β administration were evaluated in the context of hemorrhagic stroke. Microglia showed a generally dampened inflammatory profile when TGF-β was directly injected into the brains of mice with induced intracerebral hemorrhage, and the treated mice underwent a quicker functional recovery [16]. Therefore, TGF-β may be a strong candidate as a future adjunctive therapy in the treatment of acute brain injury, especially in bacterial meningitis. We performed Nissl staining to identify the therapeutic effects of TGF-β administration in bacterial-meningitis-induced brain damage. The results show that fewer shrunken neurons with pyknotic nuclei were found in mice that received NMEC with TGF-β via the tail vein, and TGF-β did not affect the development of bacteremia (Figure 4a and Figure S4), meaning that TGF-β could be a strong candidate as a future adjunctive therapy in the treatment of bacterial meningitis.

## 3. Discussion

**Figure 5:**
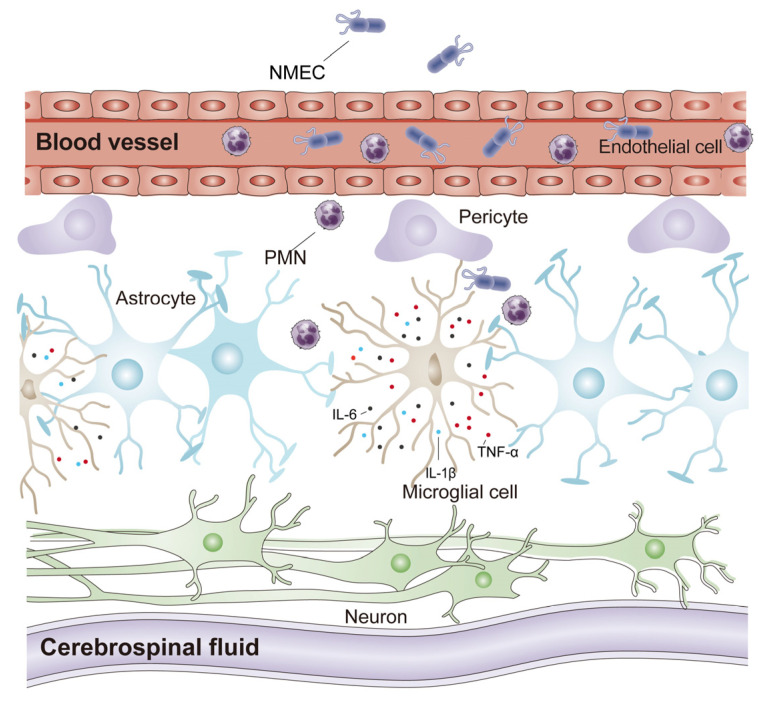
Model of the signaling pathway in microglia activation during NMEC infection.

Meningitis-causing pathogens exhibit the specific trait of penetration of the BBB and cause mortality and morbidity worldwide, as well as many irreversible sequelae [17]. Our incomplete knowledge of their pathogenesis contributes to such mortality and morbidity. Early antibiotic treatment improves outcomes, but the emergence and growth of drug resistance, especially in *E. coli*, has created a challenge and is fueling the further development of new vaccines and treatment strategies. Here, we found that NMEC infection induces the activation of microglia towards the M1 state and the production of inflammatory factors. The secretion of inflammatory factors is a double-edged sword that promotes PMN recruitment to the brain to clear pathogens but contributes to brain damage at the same time (Figure 5). We also found that TGF-β reduces NMEC-caused inflammation-related neuronal death in an animal model, and it may be useful as an adjunctive therapy to help develop novel strategies for bacterial meningitis treatment.

Microglia are the resident macrophages of the CNS, which, when activated, can trigger a host of immunological pathways. Classical activation increases the production of pro-inflammatory cytokines, chemokines and reactive oxygen species and is regarded as the M1 phenotype, while alternative activation is implicated in the inhibition of inflammation and restoration of homeostasis, and is regarded as the M2 phenotype [18]. The inflammatory response of the M1 phenotype can facilitate the elimination of invasive microorganisms. However, the M2 phenotype is associated with the capacity to downregulate inflammation and promote tissue repair. As the results of RNA-seq analyses of microglia in vitro and in vivo show, microglia are activated via NMEC infection to produce inflammatory factors. Increasing evidence suggests that the recruitment of a large number of lymphocytes in the CNS may promote the occurrence and progression of inflammation in nervous system diseases [19]. We found that the secretion of inflammatory factors by microglia promotes PMN recruitment to the CNS. Previous studies also showed that excessive or extended microglial activation can result in neuronal damage and eventually cell death [20]. As the results of Nissl staining show, NMEC infection induces more shrunken neurons with pyknotic nuclei and contributes to brain damage. Although there was a limitation in this study in that microglia and astrocytes are the main sources of inflammatory factors and we found that IL-6 is mainly produced by microglia, they are not the only producers of IL-6; other cells such as dendritic cells, neurons and endothelial cells can also produce IL-6 [21,22,23,24]. Therefore, the intracranial inflammatory response caused by NMEC needs further study in the future.

The high morbidity, mortality and sustained neurological sequelae promoted the investigation of several adjunctive therapies in animal models, of which the common goal is to reduce inflammation-related neuronal death and brain damage. However, they have shown poor performance in subsequent clinical trials thus far [25]. Previous research showed that TGF-β plays an important role as an anti-inflammatory cytokine. In microglia, this cytokine inhibits phagocytosis [26] and the production of TNF-α, IL-1β and IL-6 [27,28]. TGF-β was also previously shown to inhibit the occurrence of kidney inflammation by activating the Smad pathway in renal fibrosis [29]. We performed Nissl staining to identify the potential therapeutic effects of TGF-β administration in bacterial-meningitis-induced brain damage. As the results show, administration of TGF-β to mice reduced shrunken neurons with pyknotic nuclei, which means that TGF-β may be a strong candidate as a future adjunctive therapy in the treatment of acute bacterial meningitis. At present, there are few reports about the treatment of bacterial meningitis by intracranial injection of TGF-β. As such there is a need for further studies and these studies should be designed to detect a relevant clinical outcome, which is convincing enough to justify a clinical trial. Although further research is needed, our findings provide new insights into the treatment of bacterial meningitis.

## 4. Materials and Methods

### 4.1. Cell Line

N9 cells were cultured in DMEM (Life Technologies Corporation, New York, NY, USA) supplemented with 10% fetal bovine serum (FBS) (Genetimes ExCell International Holdings Limited, Hong Kong, China), 100 U/mL penicillin and 100 mg/mL streptomycin. The cells were incubated at 37 °C in a humidified atmosphere containing 5% CO_2_.

### 4.2. Animal Model

Five-day-old BALB/c mice (Beijing Vital River Laboratory Animal Technology Co., Ltd., Beijing, China) were used for collection of cerebrospinal fluid (CSF) samples after intracranial injection of NMEC (2.5 × 10^3^ CFUs) and with or without 25 ng/mouse LPS [30,31,32]. The collected CSF (diluted with PBS or diluent 1:100) was used for counting NMEC colonies or quantifying MPO and IL-6 protein levels via ELISA.

Eighteen-day-old BALB/c mice (Beijing Vital River Laboratory Animal Technology Co., Ltd.) were used for collecting brain tissue after tail-vein injection of NMEC (1 × 10^7^ CFUs) [33] and with or without 5 μg/mouse TGF-β (Sino Biological, Beijing, China) [34]. Brain tissues were used for brain-slice immunofluorescence.

All mice were housed in a specific-pathogen-free (SPF) mouse facility. All experiments were conducted in accordance with ethical guidelines and approved by the Animal Care and Use Committee of Nankai University, Tianjin, China.

### 4.3. Bacterial Strains

The strain used in this study was derived from a clinical isolate, RS218 (serotype O18:K1:H7), which was initially isolated from the cerebrospinal fluid of a newborn with meningitis in San Francisco and provided by Kwang Sik Kim (Johns Hopkins University, Baltimore, MD, USA) [35].

### 4.4. NMEC Infection of N9 Microglial Cells

Overnight cultures were subcultured at 1:100 in LB with appropriate antibiotics and incubated in a shaking incubator (37 °C, 180 rpm) to reach exponential growth phase. NMEC was resuspended in DMEM and added in a multiplicity of infection of 100:1 to N9 cells, which were incubated at 37 °C in a 5% CO_2_ incubator for 90 min for infection assays. The cells were washed three times with PBS and collected for RNA extraction and transcriptome analysis.

### 4.5. Isolation of Microglia from Mice

Eighteen-day-old BALB/c mice received NMEC (1 × 10^7^ CFUs) or 1 × PBS as control via tail-vein injection. Four hours later, mice were transcardially perfused with 20 mL of 0.9% NaCl for 10 min. Brain tissues were collected and microglia were isolated using the EasySep Mouse CD11b Positive Selection Kit (Stemcell Technologies, Vancouver, BC, Canada) according to the manufacturer’s instructions. The microglia were used for RNA extraction and transcriptome analysis.

### 4.6. RNA-seq Library Construction and Sequencing

The total RNA of the microglia was extracted using NucleoSpin RNA Plus XS kit (Macherey-Nagel, Düren, North Rhine-Westphalia, Germany) according to the manufacturer’s instructions. Three biological replicates were carried out for each condition. RNA libraries were generated using NEB Next Ultra RNA Library Prep Kit and sequenced on an Illumina HiSeq platform, and 125 bp/150 bp paired-end reads were generated.

### 4.7. RNA-seq Data Analysis

Raw reads in fastq format were first processed through in-house Perl scripts. In this step, clean data (clean reads) were obtained by removing reads containing adapter, reads containing ploy-N and low-quality reads from the raw data. At the same time, the Q20, Q30 and GC content of the clean data were calculated. Reference genome and gene model annotation files were downloaded directly from the genome website. The reference genome index was built using Hisat2 v2.0.5 to align the paired-end clean reads with the reference genome. The statistical software R (v3.3.2, https://www.r-project.org/, accessed on 16 September 2021) and Bioconductor(v3.4, http://www.bioconductor.org/, accessed on 16 September 2021) packages were applied to the significance analysis of differently expressed genes (DEGs) between the NMEC infection group and control group. The biological significance of DEGs was explored via Gene Ontology (GO) term enrichment analysis based on the Bioconductor package “GOstats”. Kyoto Encyclopedia of Genes and Genomes (KEGG) pathway enrichment analysis of DEGs was performed using the Bioconductor package “GeneAnswers” to find critical pathways. *p* < 0.05 was considered to have statistical significance and to achieve significant enrichment.

### 4.8. Brain Slice Immunofluorescence

Eighteen-day-old BALB/c mice were injected with 1 × 10^7^ NMEC CFUs via the tail vein. Four hours later, the mice were perfused, and the brain samples were collected. Tissue sections of 4 mm thickness were prepared on a Leica Cm1950 cryostat platform (Leica Microsystems Co., Ltd., Oakland, CA, USA). The slices were fixed in ice-cold 4% paraformaldehyde (PFA) for 10 min and sealed with 5% BSA for 1 h. The slices were incubated with primary antibody in 5% BSA at 4 °C overnight, followed by staining with fluorescently labeled secondary antibodies in 5% BSA for 1 h at room temperature. Nuclei were stained with DAPI (4′,6-diamidino-2-phenylindole, (Life Technologies Corporation, New York, United States). The primary antibodies involved were Iba1 (diluted 1:200, Abcam, ab48004, Cambridge, UK)), GFAP (diluted 1:200, Abcam, ab7260, Cambridge, UK)), Homer (diluted 1:200, Abcam, ab184955, Cambridge, UK)), Bassoon (diluted 1:100, Abcam, ab306593, Cambridge, UK)), IL-6 (diluted 1:100, Abcam, ab233706, Cambridge, UK)) and Ly6G (diluted 1:100, Abcam, ab238132, Cambridge, UK)). The secondary antibodies involved were Goat Anti-Rat IgG H&L (FITC) (diluted 1:200, Abcam, ab6730, Cambridge, UK)), Goat Anti-Rabbit IgG H&L (Alexa Fluor^®^ 594) (diluted 1:200, Abcam, ab150080, Cambridge, UK)) and Goat Anti-Rabbit IgG H&L (Alexa Fluor^®^ 647) (diluted 1:200, Abcam, ab150115, Cambridge, UK)). Each experiment was repeated at least three times.

### 4.9. Nissl Staining Experiment

The brain slices were prepared in the above experimental steps using a Nissl-staining kit (KGMP0185, Jiangsu Kaiji Biotechnology Co., Ltd., Nanjing, China) according to the manufacturer’s instructions, and a Pannoramic Confocal (MSHOT 3DHISTECH, 3DHISTECH P250 FLASH, Budapest, Hungary) slide scanner was used for observation and analysis.

### 4.10. Quantitative Real-Time PCR

qRT-PCR analysis was carried out on an ABI 7500 real-time PCR system (Applied Biological Systems, Franklin Lakes, NJ, USA). qRT-PCR was performed to verify that the changes in gene expression were detected using transcriptome analysis. The primer sequences are listed in Table 1. Each qRT-PCR was carried out in a 96-well optical reaction plate (Applied Biosystems, New York, NY, USA) with a total volume of 20 μL, which contained 10 μL Power SYBR Green PCR Master Mix (Applied Biosystems, New York, USA), 1 μL cDNA and two gene-specific primers; the *Gapdh* gene was used as a standardized reference control. The relative difference in gene expression was calculated using the 2^−ΔΔCt^ method, which calculates the relative multiple in gene expression between the experimental sample and the control sample [36]. Ct represents the cycle threshold of the sample produced by qRT-PCR; ΔCt = Ct (target gene) − Ct (*Gapdh*), ΔΔCt = ΔCt (experimental sample) − ΔCt (control sample). At least three biological repeats for each qRT-PCR analysis were established.

### 4.11. Enzyme-Linked Immunosorbent Assay (ELISA) Experiment

Five-day-old BALB/c mice received 2.5 × 10^3^ CFUs intracranially. After 6 h, CSF samples were collected and diluted with PBS or diluent 1:100 [30,31]. The levels of MPO (RK00385, Abclonal, Boston, MA, USA) and IL-6 (D721175, Sangon Biotech, Shanghai, China) were quantified. Experiments were performed according to the protocols of the manufacturer.

### 4.12. Statistical Analyses

All experimental data were analyzed using GraphPad software (version 7.0; GraphPad Software, La Jolla, CA, USA). The data presented in each figure are mean values ± standard deviation (SD) from three independent experiments. Differences between two mean values were evaluated using a two-tailed Student’s *t*-test. For mouse experiments, statistical significance was assessed via the Mann–Whitney U test. In all cases, *p* < 0.05 was considered significant; ns represents no significant difference.

## Figures and Tables

**Table 1:** The qRT-PCR primer sequences.

Primer	Sequence
*il6*	AGGATACCACTCCCAACAGACCT CAAGTGCATCATCGTTGTTCATAC
*il1b*	TGAGGACATGAGCACCTTCTT GTTCATCTCGGAGCCTGTAGT
*Gapdh*	AACTCCCACTCTTCCACCTTC GGTCCAGGGTTTCTTACTCCTT

## Data Availability

All data are presented within the manuscript and the Supplementary Materials.

## Supplementary Materials

The supporting information can be downloaded at: https://www.mdpi.com/article/10.3390/ijms24129915/s1.
