# Supplementary material for: Neonatal Meningitis-Causing Escherichia coli Induces Microglia Activation which Acts as a Double-Edged Sword in Bacterial Meningitis
Source: Int J Mol Sci. 2023 Jun 8;24(12):9915. doi: 10.3390/ijms24129915 (PMC10298365; doi:10.3390/ijms24129915)
Supplement: Supplementary file 1 [file ijms-24-09915-s001.zip › ijms-2353607-SI.pdf]

Neonatal meningitis–causing *Escherichia coli* induces microglia activation which acts as a double–edged sword in bacterial meningitis

Supplementary Material

Yingying Su[1,2], Guozhen Ma[1,2], Yangyang Zheng[1,2], Jingliang Qin[1,2], Xiaoya Li[1,2], Qianwen Ge[1,2], Hao Sun[1,2,*] and Bin Liu[1,2,*]

1. The Key Laboratory of Molecular Microbiology and Technology, Ministry of Education, Nankai University, Tianjin 300457, China

2. Tianjin Key Laboratory of Microbial Functional Genomics, TEDA Institute of Biological Sciences and Biotechnology, Nankai University, Tianjin 300457, China

* Author to whom correspondence should be addressed.

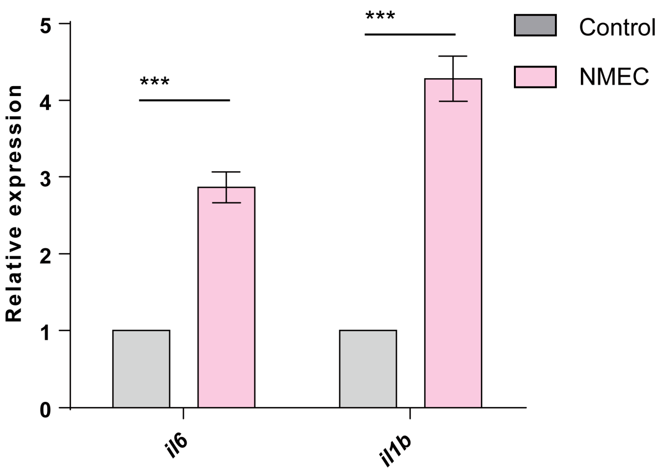

Figure S1. qRT−PCR analysis of *il6* and *il1b* mRNA levels in untreated microglia (control) or microglia infected with NMEC. n = 3 independent in vitro experiments. P values were determined using the two−tailed unpaired Student’s *t*−test. *** represents $P < 0.001$.

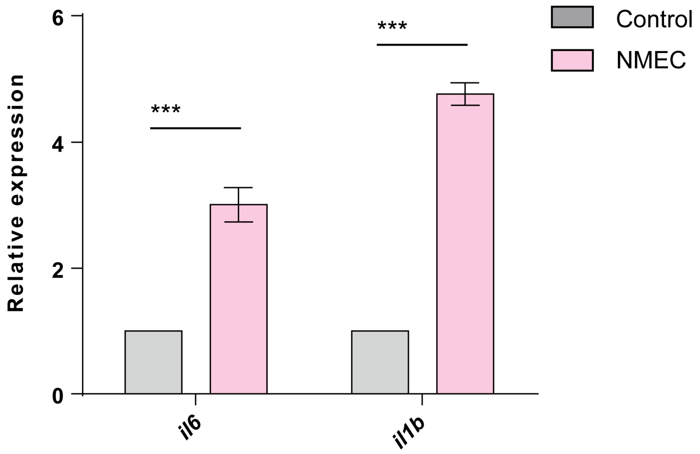

Figure S2. qRT–PCR analysis of *il6* and *il1b* mRNA levels in microglia isolated from mice that received PBS (control) or NMEC. n = 3 independent in vitro experiments. P values were determined using the two – tailed unpaired Student's *t*–test. *** represents P < 0.001.

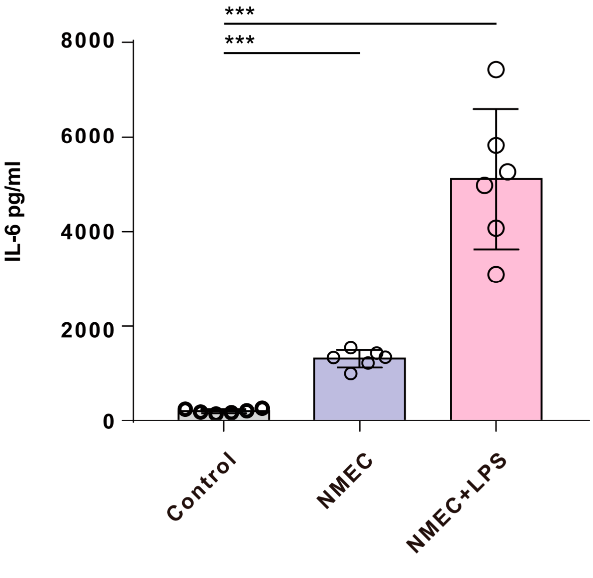

Figure S3. ELISA quantification of IL–6 levels in CSF from mice that received PBS (control) or NMEC with or without LPS intracranially. P values were determined using the Mann–Whitney U test. *** represents P < 0.001.

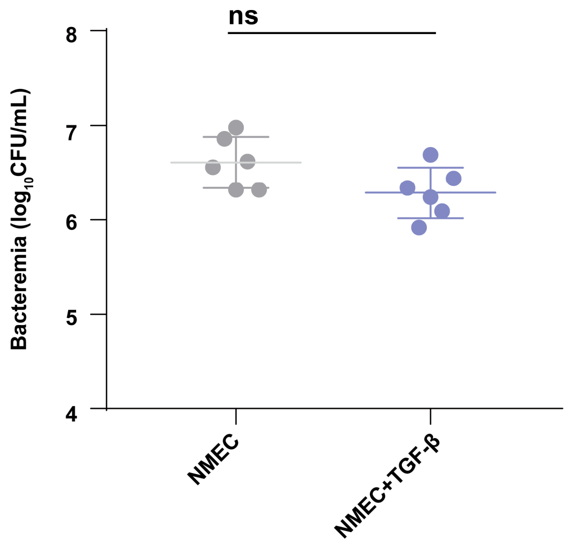

Figure S4. Bacterial counts in the blood of mice that received NMEC with or without TGF–β; ns represents no significant difference.
